# Adenosine deaminase acting on RNA 1 (ADAR1) as crucial regulators in cardiovascular diseases: structures, pathogenesis, and potential therapeutic approach

**DOI:** 10.3389/fphar.2023.1194884

**Published:** 2023-08-17

**Authors:** Jieying Chen, Junyan Jin, Jun Jiang, Yaping Wang

**Affiliations:** ^1^ Department of Cardiology of The Second Affiliated Hospital, School of Medicine Zhejiang University, Hangzhou, China; ^2^ Cardiovascular Key Laboratory of Zhejiang Province, Hangzhou, China

**Keywords:** ADAR1, RNA editing, target gene regulation, potential therapeutic approach, cardiovascular diseases

## Abstract

Cardiovascular diseases (CVDs) are a group of diseases that have a major impact on global health and are the leading cause of death. A large number of chemical base modifications in ribonucleic acid (RNA) are associated with cardiovascular diseases. A variety of ribonucleic acid modifications exist in cells, among which adenosine deaminase-dependent modification is one of the most common ribonucleic acid modifications. Adenosine deaminase acting on ribonucleic acid 1 (Adenosine deaminase acting on RNA 1) is a widely expressed double-stranded ribonucleic acid adenosine deaminase that forms inosine (A-to-I) by catalyzing the deamination of adenosine at specific sites of the target ribonucleic acid. In this review, we provide a comprehensive overview of the structure of Adenosine deaminase acting on RNA 1 and summarize the regulatory mechanisms of ADAR1-mediated ribonucleic acid editing in cardiovascular diseases, indicating Adenosine deaminase acting on RNA 1 as a promising therapeutic target in cardiovascular diseases.

## 1 Introduction

Cardiovascular diseases (CVDs) represent the number one cause of death globally, accounting for nearly 50% of the 36 million deaths from non-communicable diseases worldwide each year (DALYs and HALE Collaborators, 2018). Research on the genetic level is helpful for better prevention and treatment of CVDs (Lu et al., 2023). Accumulating evidence indicates that ribonucleic acid (RNA) editing plays a crucial role in the initiation and progression of CVDs and may be a potential therapeutic strategy (Birgaoanu et al., 2022). RNA editing refers to the deletion, addition, and modification of nucleotides in the coding region of post-transcriptional RNA to change genetic information. It is an indirect and highly specific repair mechanism for genetic material and widely exists in biological organisms (Sun et al., 2016). RNA-dependent adenine riboside deaminase plays integral roles in various post-transcriptional regulatory processes (Wang et al., 2017b). ADAR1 belongs to adenosine to inosine (A-to-I) RNA editing enzyme, which is a specific double-stranded RNA adenosine deaminase. Its main function is RNA editing, inducing nuclear protein translation, which converts precursor messenger RNA (pre-specific adenosine in mRNA) to inosine (Song et al., 2022). ADAR1 is involved in a variety of important physiological processes, including embryonic development, hematopoietic system development, central nervous system transmitter transmission, and body immunity, etc (Hartner et al., 2004; Wang et al., 2004; Jacobs et al., 2009; XuFeng et al., 2009; Niescierowicz et al., 2022). A lot of attention has been paid to ADAR1 since its discovery, particularly in CVDs. A-to-I RNA editing has been found significantly elevated in patients with CVDs (Stellos et al., 2016; Vlachogiannis et al., 2021). Here, we review existing literature on RNA editing mechanism of ADAR1 and the pathophysiology of the cardiovascular system and discuss implications and outstanding questions in the field.

## 2 ADAR1 and its domain structures

ADAR1, ADAR2 and ADAR3 are the three most conserved members of the mammalian ADAR family (Nakahama and Kawahara, 2020). ADAR1 is ubiquitous in nature and performs A-to-I RNA editing at millions of sites in the human transcriptome (Raghava Kurup et al., 2022). ADAR1 is highly expressed in fetal and adult hearts and in blood vessels, and ADAR1 is critical for maintaining cardiac homeostasis and function in the adult heart (El Azzouzi et al., 2020). Compared with ADAR1, the expression of ADAR2 is restricted and is highest in the brain and central nervous system (Tan et al., 2017). Although Jain et al. found higher levels of ADAR2 expression in tibial arteries, aorta, coronary arteries, and other vascular tissues (Jain et al., 2018), ADAR2 is generally less expressed than ADAR1 in peripheral tissues, including the heart (Tan et al., 2017). ADAR2 is thought to function primarily in the brain, and its clinical significance has been associated with neurologically-related functions so far, such as aminomethylphosphonic acid receptor editing, which, if disturbed, can lead to intractable seizures and death (Jain et al., 2018). ADAR1 and ADAR2 form respective homodimers, and this characteristic is critical for their enzymatic activity (Thuy-Boun et al., 2020). It is worth mentioning that the expression of ADAR3 is limited to the brain (Melcher et al., 1996), cannot homodimerize, and has no catalytic activity. The main biological function lies in its ability to bind to other ADAR competing RNA, including participating in regulating the editing effect of ADAR2 (Mladenova et al., 2018; Raghava Kurup et al., 2022).

The general structure of ADAR protein includes the N-terminal double-stranded RNA binding domain and the C-terminal deaminase domain that catalyzes the substrate reaction (Goodman et al., 2012). ADAR1 is the most extensively investigated RNA editing enzyme with various biological functions (Kim et al., 1994; Wang et al., 2017b). Chromosome ADAR1 gene is located on 1q21.1-21.2, total length of about 40 kb, including 17 exons (Weier et al., 1995). The N-terminus of ADAR1 has three double-stranded RNA (dsRNA)-binding domains, while the C-terminus has a conserved catalytic deamination domain. The N-terminus also possesses a Z-DNA binding domain that binds nascent RNA and undergoes pre-splicing modification (Wang et al., 2017a). The biological function of ADAR1 mainly includes RNA editing, that is, converting adenosine in dsRNA substrate to inosine at the post-transcriptional level. Inosine (I) is recognized by ribosomes and RNA polymerase as guanosine (G), thus leading to the replacement of RNA base A→G during transcription (Samuel, 2019). If base editing occurs in non-coding regions, it may affect the stability of RNA and thus alter the expression of subsequent proteins. But once it occurs in the coding region, the sequence change will directly affect the coded codon and thus change the amino acid sequence of the protein (Picardi et al., 2015). Most of these specific sites in human cells are found in the non-coding parts of the transcriptome, such as introns and untranslated regions (3′-UTR) (Tan et al., 2017; Eisenberg and Levanon, 2018).

**FIGURE 1:**
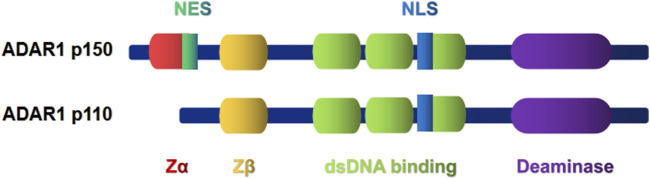
ADAR1 protein has three domains (light green) that bind to dsRNA and a conserved catalytic deamination domain (purple) at the C-terminal. The difference is that ADAR1 p150 contains a Z-α domain (red), called Z-RNA, that binds to left-handed dsRNAs. The Zβ domain (yellow), which is homologous to the Zα structure, is separated from the Zα by a long linker at the N terminal of ADAR1, but its function remains unknown. ADAR1 p110 lacks the Z-α domain. In addition, most of the Adarl p150 was localized in the cytoplasm due to a nuclear export signal (NES; shown in green) segment in the Z-DNA binding domain near the N terminal. The nuclear localization signal (NLS; shown in blue) is present in both p150 and p110 isomers.

There are two subtypes of ADAR1, the full-length p150 and the shorter p110 (Patterson and Samuel, 1995). The structure and function of the two subtypes are different (Figure 1). ADAR1 p150 is induced by interferon (IFN) and contains deoxyribonucleic acid (DNA)/RNA binding domains (Zα and Zβ domains). Only the Zα domain has Z-DNA binding ability (Athanasiadis et al., 2005). ADAR1 p150 is mostly located in the cytoplasm due to an nucleation signal near the N-terminal Z-DNA binding domain (Ng et al., 2013). In addition, compared with p150, the domain of p110 subtype lacks NES and Zα domain. So almost all of p110 subtype is localized in the nucleus (Sun et al., 2021). Z-DNA binding protein 1 (ZBP1) is currently found to be the only protein containing a Zα domain in mammals (Schwartz et al., 2001). Recent focus has been on ADAR1 regulating aberrant immune activation through the canonical A-to-I RNA editing pathway. Loss of ADAR1 function can activate ZBP1-mediated autoimmune diseases and embryonic death, suggesting that ADAR1 is inversely associated with immune activation (de Reuver et al., 2022; Hubbard et al., 2022; Jiao et al., 2022). Recognition of Alu duplex RNA by ZBP1 may contribute to the ADAR1 pathological features of loss-of-function Aicardi–Goutières syndrome (de Reuver et al., 2022). Meanwhile, ZBP1-dependent signaling underlies autoinflammatory pathology induced by ADAR1 alterations (Hubbard et al., 2022). The role of ADAR1 in innate immunity has been apparent for many years. ADAR1 can regulate innate immunity by inhibiting the pattern recognition receptor mechanism, and activation of ADAR1 inhibits IFN expression and IFN-mediated antiviral activity (Li et al., 2016). During virus infection, the long-chain exogenous dsRNA is recognized by melanoma differentiation-associated gene 5 (MDA5), protein kinase R (PKR) and oligoadenylate synthetase (OAS), which initiates three pathways leading to cell apoptosis to prevent virus replication (Quin et al., 2021). In addition to immune-related diseases, ADAR1 is also involved in the immune recognition of tumors (Zhang et al., 2018). Zhang et al. found that ADAR1 represses endogenous Z-RNAs and identified ZBP1-mediated necroptosis as a novel determinant of ADAR1-masked tumor immunogenicity (Zhang et al., 2022). But in terms of clinical translation, the current lack of specific small molecule inhibitors of ADAR1 limits the development of therapeutic drugs for the treatment of tumors and autoimmune diseases, which will be urgently needed in the future.

The ADAR2 protein encodes two major isoforms: ADAR2S (or ADAR2a) and ADAR2L (or ADAR2b) (Gerber et al., 1997). ADAR2 isoforms differ most structurally from ADAR1 in the number of dsRNA-binding domains and the amino-terminal extension of the ADAR enzyme. ADAR2 has only two dsRNA-binding motif repeats in the N-terminal region, has no Z domain, and is not regulated by IFN (Gerber et al., 1997). Compared with ADAR1, ADAR2 is more conserved, and its homologous sequence can be found in the *drosophila* genome (Heraud-Farlow and Walkley, 2020).

In general, ADAR1 is mainly responsible for the editing of repetitive sequences (such as primate Alu), and ADAR2 is mainly responsible for the editing of messenger RNA (mRNA) coding regions, performing site-specific editing (Tan et al., 2017). Isomers of ADARs have potentially different activities, which may include different substrate specificities, different subcellular localizations, and different interactions with proteins or the ability to bind different nucleic acids (Samuel, 2012). Kokot et al. found that the expression of ADAR1 was increased and the expression of ADAR2 was decreased in the failing heart. Among them, ADAR2 underwent proteasomal degradation in human cardiomyocytes, the depth of A-to-I RNA editing was reduced, and the expression of circular RNA (circRNA) was increased (Kokot et al., 2022). Recent studies have found that ADAR2 is not a key factor in heart development, but may have a cardioprotective function as it regulates the expression of microRNAs(miRNAs) and their corresponding targets (Altaf et al., 2019). Research about ADAR2 on CVDs is ongoing, and future research may help decipher the link between CVDs and ADAR2 activity. Therefore, in this review, we mainly focus on the pathophysiological process of ADAR1 in cardiovascular aspects.

## 3 RNA editing activity of ADAR1 and cardiovascular system

RNA editing is performed by A-to-I editors, which are members of the ADAR protein family (Christofi and Zaravinos, 2019). The regulation of A-to-I editing is complex, and the influencing factors include ADAR expression and localization, downstream RNA target selection, and other intermediate regulatory factors such as RNA-binding proteins (Eggington et al., 2011; Ramaswami and Li, 2014). Jain et al. found increased vasoconstrictive and diastolic hypertension was observed with reduced ADAR-mediated RNA editing in mouse models, ultimately leading to reduced cardiac remodeling and systolic output (Jain et al., 2018). The example demonstrates a causal relationship between RNA editing and the development of CVDs, suggesting that RNA modification can maintain cardiovascular health.

A-to-I editing not only occurs in the protein coding region of mRNAs, but also occurs in the non-coding region, especially the Alu region (Nishikura, 2016). Cathepsin S is a cysteine protease associated with angiogenesis. Inhibition of cathepsin S is an effective way to reduce atherosclerotic vascular diseases (Ni et al., 2020). ADAR1-mediated RNA editing is required for cathepsin S (CTSS) expression during atherosclerotic cardiovascular disease (ASCVD), aligning the 3′untranslated region (3′UTR) of the transcript with RNA-binding protein (RBP) combined more tightly, so that the structure of RNA is uneasy to be degraded, and the translation increases (Stellos et al., 2016).

MiRNA are short non-coding RNA molecules that regulate gene expression at the post-transcriptional level (Dexheimer and Cochella, 2020). MiRNA is associated with many pathophysiological processes of CVDs, including cardiac remodeling, myocardial hypertrophy, myocardial fibrosis, etc (Hata, 2013). The effect of ADAR1 on miRNA is multifaceted. On the one hand, ADAR1 can edit miRNA and its precursor pre-miRNA (Wang and Liang, 2018). On the other hand, ADAR1 can act on miRNA through non-RNA editing pathway, and the pre-miRNA maturation process requires Drosha and Dicer to participate in the shearing. ADAR1 can promote miRNA production by binding to the DUF283 domain of Dicer and the N-terminal of DEAD-box RNA helicase domain (Ota et al., 2013), and can also reduce miRNA production by inhibiting Drosha binding to target RNA (Bahn et al., 2015). Ota et al. found a different interaction between RNA editing mechanisms and RNAi mechanisms. ADAR1 forms complexes with Dicer, promoting miRNA processing and RNA-induced silencing complex (RISC) loading of miRNA, thus promoting silencing of target RNA, and thus causing stimulative effect on RNAi, rather than antagonistic effect (Ota et al., 2013). MiRNA plays a key role in various aspects of cardiovascular development and disease processes (Hata, 2013), so the regulatory effect of ADAR1 on CVDs is amplified.

In addition to miRNA, long noncoding RNA (lncRNA) is also the transcription product of non-coding genes, which is usually more than 200 nucleotides (nt) in length (Marques and Ponting, 2009). RNA editing of lncRNAs has important implications for human ASCVD and lncRNA stability, modulating gene expression of pro-inflammatory transcripts. In ASCVD, ADAR1 edits nuclear paraspeckle assembly transcript 1(NEAT1) lncRNA, promotes the recruitment of AU-rich RNA-binding factor 1 (AUF1), and controls lncRNA stability (Vlachogiannis et al., 2021).

## 4 ADAR1 in CVDS

### 4.1 ADAR1 in heart failure

Heart failure is characterized primarily by the inability of the heart to provide metabolic needs and organ/tissue perfusion (Tsao et al., 2022). Some common stimuli including oxidative stress, hypoxia, and ischemic injury can induce ER stress, which triggers unfolded protein response (UPR) activation and leads to heart failure (Liu and Dudley, 2014). ADAR1 is essential for maintaining cardiac homeostasis and function in adult hearts through complex mechanisms involving miRNA and endoplasmic reticulum stress processing (El Azzouzi et al., 2020). Moore et al. found that a tamoxifen-induced ADAR1 knockout cardiomyocyte model developed acute heart failure. RNA-sequencing identified upregulation of apoptotic genes, decreased trabecular compaction, and increased numbers of apoptotic cardiomyocytes (Moore et al., 2020). Cardiomyocyte specific depletion of ADAR1 in adult hearts activated an UPR-driven apoptotic response, and reduced miR-199a-5p levels lead to severe ventricular remodeling and rapid spontaneous cardiac dysfunction, which was reversed by restoration of miRNA expression (El Azzouzi et al., 2020). Excessive or persistent activation of the cellular innate immune response can cause to worsening and/or chronic inflammatory processes, eventually leading to heart failure (Huang et al., 2009). The loss of ADAR1 initially recruits inflammatory cells and remodeled cardiomyocytes, gradually progressing to heart failure. At the same time, Garcia-Gonzalez et al. found that in the absence of ADAR1-mediated RNA editing, IRF7 inactivation prevented heart failure by blocking the stimulation of autoinflammatory processes (Garcia-Gonzalez et al., 2022). In addition, Crow et al. first reported three cases of children with biallelic ADAR mutations. Their hearts showed calcified valvular heart disease. As the disease progresses, symptoms of biventricular failure appeared. Despite the most rigorous treatment, they died of fatal heart failure (Crow et al., 2020).

### 4.2 ADAR1 in congenital heart disease

Congenital heart disease (CHD) is currently the most common type of birth defect in the general population with an incidence of about 1% (Linglart and Bonnet, 2022). Interestingly, few studies have identified the role of RNA editing in CHD. The rate of A-to-I RNA editing was significantly higher in children with cyanotic congenital heart disease than in children without cyanosis (Borik et al., 2011). Posttranscriptional RNA changes may affect cellular and metabolic pathways, thus affecting perioperative processes following hypoxia (Borik et al., 2011). The level of ADAR2 RNA was significantly decreased in cyanotic congenital heart disease patients, while the expression of ADAR1 showed no significant difference (Borik et al., 2011). Studies have found that the expression level of ADAR2 was lower in rats under hypoxia after cerebral ischemia. Therefore, reduced ADAR2 levels in patients with cyanosis may also be associated with hypoxemia (Peng et al., 2006). Altaf et al. observed strong downregulation of ADAR2 and increased expression of ADAR1 in blood samples from patients with CHD (Altaf et al., 2019). In detail, high expression of two subtypes of ADAR1 (p150 and p110) was observed in the atrial tissues of patients with atrial septal defect and ventricular septal defect (VSD), and ADAR1 expression was increased approximately threefold in patients with tetralogy of fallot and atrioventricular septal defect compared to the control group (Altaf et al., 2019). Liu et al. reported the first Chinese case of dyschromatosis symmetrica hereditaria with Aicardi-Goutières syndrome caused by A homozygous mutation of the ADAR1 gene (c.1622T>A). In cardiac terms, the patient had significant cardiac involvement, patent ductus arteriosus, VSD, and symptoms of progressive heart failure (Liu et al., 2022). It may be related to the mutation site located in the protein domain of ADAR1 gene (Liu et al., 2022).

### 4.3 ADAR1 in atherosclerosis

A-to-I-Alu RNA editing is critical for pro-inflammatory gene expression in vascular cells during atherosclerosis (Stellos et al., 2016). ADAR1-mediated RNA editing is dynamically regulated in human umbilical vein endothelial cells under hypoxic and pro-inflammatory conditions. The CTSS gene encodes cathepsin S, which influences atherosclerosis through the enzyme’s elastolysis and collagen-lysis activity. ADAR1 knockdown can lead to about 60% downregulation of CTSS mRNA expression, which can reduce atherosclerosis and angiogenesis *in vivo*. Specifically, the mechanism involved in regulation is that the RNA-binding protein HuR preferentially binds to the edited CTSS3 ‘UTR, thus controlling the expression of CTSS mRNA (Stellos et al., 2016). Vlachogiannis et al. found that ADAR1 promoted the binding of RNA stabilizing protein AUF1 in an RNA editing dependent manner, controlling the stability of long noncoding RNA NEAT1. It has been shown that the long subtype of NEAT1 is critical in all stages of atherosclerosis. Given that the stability of RNA edits induced pro-inflammatory transcripts (such as CTSS or NEAT1) is increased in ASCVD, increased RNA editing may aid the inflammatory process of atherosclerosis (Vlachogiannis et al., 2021). One study showed that blocking ADAR1 significantly downregulates smooth muscle cell (SMC) markers and inhibits damage-induced neointima formation, which is critical for regulating damage-induced vascular remodeling (Fei et al., 2016).

Furthermore, Kong et al. first discovered the enhanced expression of Endonuclease V (ENDOV) in human carotid plaque. In addition, the expression of ADAR1 and inosine in human carotid plaque mRNA was also enhanced. ENDOV can promote atherogenesis by enhancing the recruitment of monocytes into atherosclerotic lesions, possibly by increasing the influence of chemokine ligand 2 (CCL2) activation on these cells. Therefore, it is reasonable to speculate that the interaction between ADAR1, adenosine-inosine editing and ENDOV may play a pathogenic role in the development and progression of atherosclerosis (Kong et al., 2021).

### 4.4 ADAR1 in viral myocarditis

Viral myocarditis is a common clinical cardiovascular disease caused by viral infections, and the main pathological feature is myocardial inflammation. A large number of studies have shown that the direct damage of the virus to the cardiomyocytes and the myocardial injury caused by the excessive immune response eventually leads to cardiomyocyte dysfunction and reduced contractility (Sozzi et al., 2022). The main known function of ADAR1 is to mediate the cellular immune response to viral infection. ADAR1 has a certain effect on virus replication and can inhibit or promote virus replication (Samuel, 2011). ADAR1 is overexpressed in the myocardium of viral myocarditis (VMC) mice as well as in Coxsackie virus B3 (CVB3)-induced cardiomyocytes (Li and Xie, 2022). ADAR1 promotes the cleavage of pre-miR-1a-3p by binding to Dicer, thereby regulating the functional activity of A20 which inhibits of cardiomyocyte inflammation and apoptosis in CVB3-induced myocarditis (Li and Xie, 2022). ADAR1p150 is a double-edged sword in CVB3-induced viral myocarditis. ADAR1p150 increases viral load by inhibiting the secretion of interferon in the early stage of viral infection, and inhibits the secretion of inflammatory cytokines by inhibiting the activation of NF-κb pathway in the middle and late stages of infection (Dong et al., 2016). Previous studies have shown that ADAR1p150 promotes the maturation of miRNA-222 by forming a complex with Dicer enzyme, thereby playing a role in regulating the expression of phosphatase and tensin homolog (PTEN), ultimately producing an anti-apoptotic effect on cardiomyocytes and participating in the pathophysiological process of viral myocarditis (Zhang et al., 2019). PTEN protein is one of the important target proteins downstream of microRNA-222 (Chun-Zhi et al., 2010; Zhou et al., 2012). PTEN protein is a multifunctional tumor suppressor, its main function is mediated by its lipid phosphatase activity, and it plays a key role in pathways related to apoptosis and cell survival. Therefore, the authors speculate that ADAR1p150 inhibits the expression of PTEN protein by promoting the expression of microRNA-222, thereby alleviating the damage of cardiomyocytes caused by virus infection.

### 4.5 ADAR1 in arterial aneurysms

Aneurysm is a local lesion or injury of the arterial wall, resulting in the destruction of the structure and mechanical properties of the arterial wall. Abdominal aortic aneurysms (AAA) are due to rupture and dissection, with high morbidity and mortality (Shtrichman et al., 2012; Schermerhorn et al., 2015). The switch of SMC from a contractile phenotype to an inflammatory phenotype plays an important role in AAA (Fei et al., 2016). It has been confirmed that ADAR1 plays an important role in SMC phenotype switching and vascular remodeling (Fei et al., 2016). ADAR1 promotes the stability of matrix metallopeptidase 2 (MMP2) and matrix metallopeptidase 9 (MMP9) mRNA in SMC by interacting with the RNA-binding protein HuR, increases the expression and activity of MMP2 and MMP9, leads to elastin degradation and medial degeneration in the vessel wall, and finally forms AAA. Therefore, ADAR1 is likely to be a new potential target to prevent the occurrence and development of AAA (Cai et al., 2021).

Microarray analysis of samples from patients with aortic aneurysms found elevated levels of ADAR1 and its editing target (CTSS) (Stellos et al., 2016). At the same time, ADAR1 knockdown leads to decreased endothelial angiogenic function, partly due to the downregulation of cathepsin S impairs angiogenesis. ADAR1-mediated RNA editing regulates CTSS mRNA expression in endothelial cell function and homeostasis. It suggests that RNA modifications, particularly RNA editing, plays a role in aortic aneurysm development (Stellos et al., 2016).

### 4.6 ADAR1 in arrhythmia

Reports directly investigating ADAR1 in arrhythmia remain scarce. Previous studies have shown that the role of ADAR1 in calcium handling is not known, but ADAR1 editing of glutamate ionotropic receptor kainate type subunit 1 (GRIK1) and glutamate ionotropic receptor kainate type subunit 2 (GRIK2) has been shown to affect calcium channel permeability (Song et al., 2016). Thus, lack of ADAR1 could theoretically make cardiomyocytes inadequately calcium processed and sensitive to arrhythmias. However, Azzouzi et al. showed no signs of arrhythmia in the ADAR1 knockout mice during echocardiographic analysis (El Azzouzi et al., 2020).

### 4.7 ADAR1 in peripheral artery disease

Kwast et al. found increased expression of ADAR1 protein in the veins of patients with peripheral artery disease (PAD) and end-stage PAD. ADAR1 can edit pri-microRNA in a microRNA-specific manner. Edited microRNAs promote pro-angiogenic function of vascular cells under ischemia/hypoxia conditions. Furthermore, miRNA editing enhanced angiogenesis *in vitro* and *in vitro* (van der Kwast et al., 2020). ADAR1-mediated pre-mRNA of SMC myosin heavy chain (SMMHC) and α-smooth muscle actin (α-SMA) showed selective splicing in phenotypic regulation of SMC. ADAR1 knockdown blocked platelet-derived growth factor B(PDGF-BB) induced RNA editing/splicing and restored SMC contractility protein expression. Thus, ADAR1-mediated editing controls vascular remodeling in physiology and disease by controlling phenotypic transitions between contraction and synthesis of SMC (Fei et al., 2016).

## 5 RNA editing and therapeutic approach

RNA therapy refers to the use of RNA-based molecules to modulate biological pathways to treat specific diseases (Seo et al., 2023). Generally speaking, RNA sequence is the key to regulate the expression and activity of its target genes. Once nucleic acid chemistry and its delivery methods are in place, we can use these established methods to produce RNA drugs for new targets in a relatively short time (Kim, 2020; Kim, 2022). In 1978, Zamecnik and Stephenson first described the use of synthetic oligonucleotides to alter protein expression by Watson Crick hybridization of RNA (Zamecnik and Stephenson, 1978). In the ensuing period, antisense oligonucleotides that inhibit protein synthesis facilitated the rapid development of RNA-based therapeutics (Bennett, 2019). Entering the new century, the introduction of RNAi and the use of siRNA to silence human genes have brought huge technological innovations to the study of gene function, and have had an unpredictable impact on the fields of biology and medicine (Elbashir et al., 2001).

Based on the mechanism of RNA editing linked to the pathophysiology of the cardiovascular system, targeted therapy for CVDs is a recent research hotspot. Many genetic diseases, including CVDs, are caused by single nucleotide changes (Robert and Pelletier, 2018). Over the past decade, researchers have discovered that microRNAs help regulate CVDs, inhibiting the formation of cholesterol, the buildup of cholesterol plaques and the cell death that follows a heart attack (Madrigal-Matute et al., 2013; Churov et al., 2019; Melita et al., 2022). RNA therapy is rapidly emerging as a treatment for CVDs. Recently, Rurik et al. used mRNA lipid nanoparticles to guide the generation of CAR-T cells *in vivo* to target the repair of damaged fibrotic cardiomyocytes and restore the heart to normal function (Rurik et al., 2022). The application of RNA therapeutics to CVDs is eagerly anticipated, but given the diverse roles of RNA-modifying enzymes in cardiovascular pathophysiology, the use of these therapies in CVDs can be challenging and requires careful consideration.

## 6 Conclusion

**FIGURE 2:**
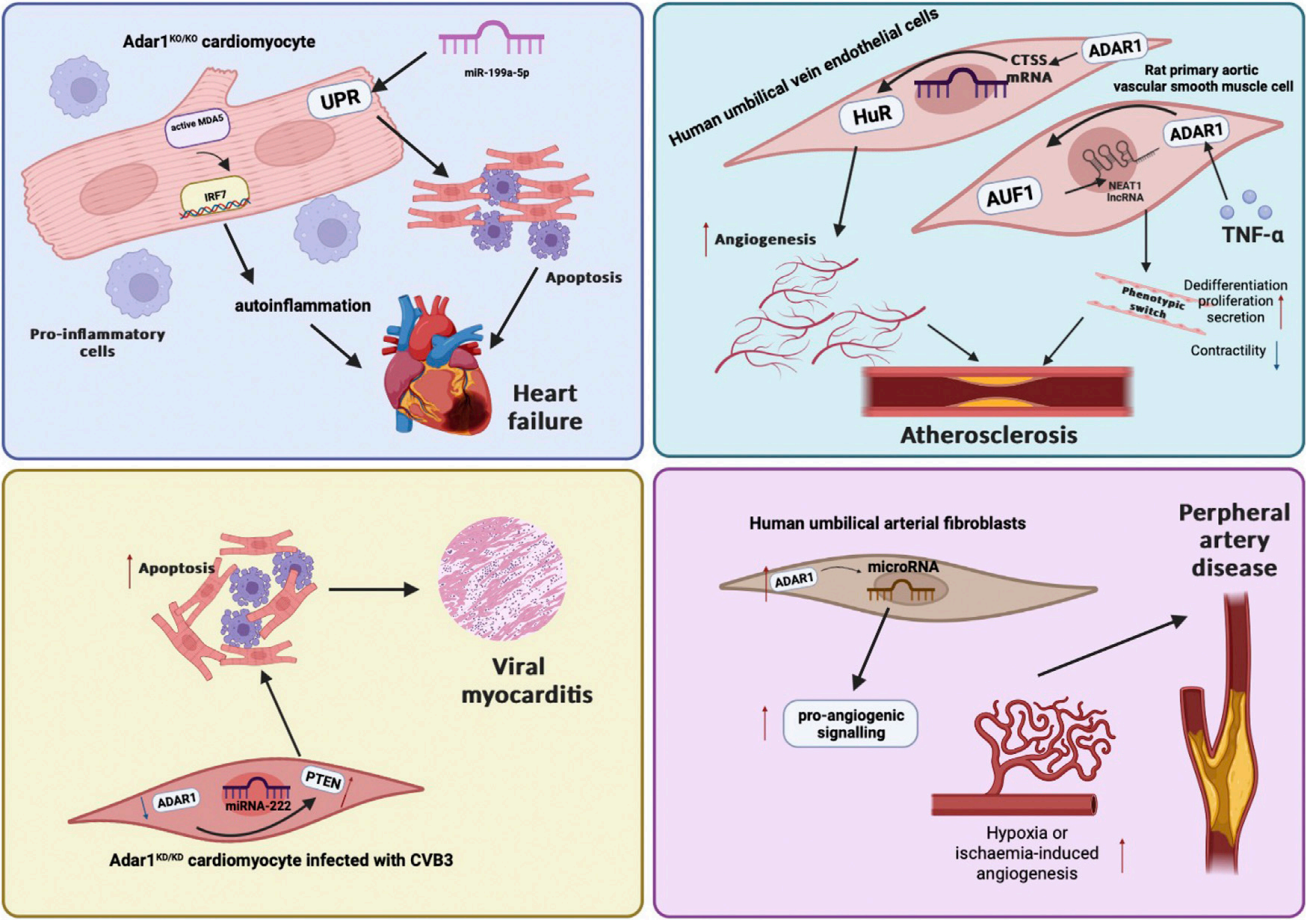
A figure about the roles of ADAR1 in cardiovascular diseases.

Cardiovascular diseases is the leading cause of death worldwide. The important point of our manuscript is that we summarize the mechanisms by which ADAR1 affects cardiac function and cardiovascular aspects as well as potential therapeutic targets (Figure 2). It is now generally recognized that ADAR1 centers on a complex balance of several layers of regulatory components, including editing events, post-translational modifications, and homo-hetero-dimer formation. Influencing factors include the expression and localization of ADAR, the selection of downstream RNA targets, and the role of other intermediate regulators such as RNA-binding proteins. So far, considerable progress has been made in the research on the regulation of the mammalian ADAR gene, the editing ability of the ADAR protein family, and the role of A-to-I editing events in the physiological and pathological processes of CVDs, but there are still many mechanisms need further research. Undoubtedly, future research needs to focus on the complexities of ADAR1 in relation to the cardiovascular domain, such as the importance of ADAR1 in other cell types in the heart, in different forms of cardiac injury (ischemia/reperfusion, pressure overload, responses to neurohumoral stimuli, diabetic cardiomyopathy, etc.), and whether ADAR1 has disturbing interactions with other organ systems during CVDs. Precise nucleic acid editing technology is expected to treat diseases from the RNA level, such as CRISPR/Cas combined with A-to-I RNA substitution editing, but these studies in CVDs have just begun. Given the differential impact of ADAR1 in different cardiovascular tissues, the use of RNA-level therapeutics for the treatment of CVDs may be challenging and requires careful consideration.

## Figures and Tables

**Table:** 

**AAA**	Abdominal aortic aneurysms
**ASCVD**	Atherosclerotic cardiovascular disease
**A-to-I**	Adenosine to inosine
**ADAR1**	Adenosine deaminase acting on RNA 1
**AUF1**	AU-rich RNA-binding factor 1
**CHD**	Congenital heart disease
**CTSS**	Cathepsin S
**CVDs**	Cardiovascular diseases
**circRNA**	Circular RNA
**CCL2**	Chemokine ligand 2
**CVB3**	Coxsackie virus B3
**dsRNA**	Double-stranded RNA
**DNA**	Deoxyribonucleic acid
**ENDOV**	Endonuclease V
**GRIK1**	Glutamate ionotropic receptor kainate type subunit 1
**GRIK2**	Glutamate ionotropic receptor kainate type subunit 2
**IFN**	Interferon
**lncRNA**	long noncoding RNA
**miRNA**	MicroRNA
**MDA5**	Melanoma differentiation-associated gene
**mRNA**	Messenger RNA
**MMP2**	Matrix metallopeptidase 2
**MMP9**	Matrix metallopeptidase 9
**NEAT1**	Nuclear paraspeckle assembly transcript
**OAS**	Oligoadenylate synthetase
**PAD**	Peripheral artery disease
**PKR**	Protein kinase R
**PTEN**	Phosphatase and tensin homolog
**PDGF-BB**	Platelet-derived growth factor
**RBP RNA**	Binding protein
**RNA**	Ribonucleic acid
**RISC RNA**	Induced silencing complex
**SMC**	Smooth muscle cell
**3′UTR**	3′untranslated region
**UPR**	Unfolded protein response
**VSD**	Ventricular septal defect
**VMC**	Viral myocarditis
**ZBP1**	Z-DNA binding protein 1
